# Cedrol, a Sesquiterpene Isolated from *Juniperus chinensis*, Inhibits Human Colorectal Tumor Growth associated through Downregulation of Minichromosome Maintenance Proteins

**DOI:** 10.15430/JCP.2023.28.2.75

**Published:** 2023-06-30

**Authors:** Soojung Jin, Jung-ha Park, Hee Jung Yun, You Na Oh, Seunghye Oh, Yung Hyun Choi, Byung Woo Kim, Hyun Ju Kwon

**Affiliations:** 1 Core-Facility Center for Tissue Regeneration, Dong-eui University, Busan, Korea; 2 Biopharmaceutical Engineering Major, Division of Applied Bioengineering, College of Engineering, Dong-eui University, Busan, Korea; 3 Department of Biopharmaceutics, Dong-eui University Graduate School, Busan, Korea; 4 Department of Biochemistry, College of Korean Medicine, Busan, Korea; 5 Blue-Bio Industry Regional Innovation Center, Dong-eui University, Busan, Korea

**J Cancer Prev 2022; 27(4):221-228**

**https://doi.org/10.15430/JCP.2022.27.4.221**

In the page 222, the forth subheading in the MATERIALS AND METHODS section, the word “vytometric” should read “cytometric.” The subheading should be corrected as follows:

**<Before correction>**

**Flow vytometric analysis of cell cycle**

**<After correction>**

**Flow cytometric analysis of cell cycle**

We apologize for our mistake and any inconvenience this may have caused.

